# A Comparative Study of Corrosion AA6061 and AlSi10Mg Alloys Produced by Extruded and Additive Manufacturing

**DOI:** 10.3390/ma14195793

**Published:** 2021-10-03

**Authors:** Francisco Estupiñan-López, Citlalli Gaona-Tiburcio, Jesús Jáquez-Muñoz, Patricia Zambrano-Robledo, Erick Maldonado-Bandala, José Cabral-Miramontes, Demetrio Nieves-Mendoza, Anabel D. Delgado, Juan Pablo Flores-De los Rios, Facundo Almeraya-Calderón

**Affiliations:** 1 Universidad Autonoma de Nuevo Leon, FIME-Centro de Investigación e Innovación en ingeniería Aeronáutica (CIIIA), Av. Universidad s/n, Ciudad Universitaria, San Nicolás de los Garza 66455, Mexico; francisco.estupinanlop@uanl.edu.mx (F.E.-L.); Jesus.jaquezmn@uanl.edu.mx (J.J.-M.); patricia.zambranor@uanl.edu.mx (P.Z.-R.); jose.cabralmr@uanl.edu.mx (J.C.-M.); 2 Facultad de Ingeniería Civil, Universidad Veracruzana, Xalapa 91000, Mexico; erimaldonado@uv.mx (E.M.-B.); dnieves@uv.mx (D.N.-M.); 3 Centro de Investigación en Materiales Avanzados (CIMAV), Miguel de Cervantes 120, Complejo Industrial Chihuahua, Chihuahua 31136, Mexico; anabel.delacruz@cimav.edu.mx; 4 Instituto Tecnológico de Chihuahua, Tecnológico Nacional de Mexico, Av. Tecnologico 2909, Chihuahua 31130, Mexico; juan.fd@chihuahua.tecnm.mx

**Keywords:** corrosion, additive manufacturing, electrochemical noise, wavelets, Hilbert–Huang, skewness, Kurtosis

## Abstract

The aim of this work was to evaluate the corrosion behavior of the AA6061 and AlSi10Mg alloys produced by extruded and additive manufacturing (selective laser melting, SLM). Alloys were immersed in two electrolytes in H_2_O and 3.5 wt. % NaCl solutions at room temperature and their corrosion behavior was studied by electrochemical noise technique (EN). Three different methods filtered EN signals, and the statistical analysis was employed to obtain Rn, the localization index (LI), Kurtosis, skew, and the potential spectral density analysis (PSD). The Energy Dispersion Plots (EDP) of wavelets method was employed to determine the type of corrosion and the Hilbert–Huang Transform (HHT), analyzing the Hilbert Spectra. The result indicated that the amplitude of the transients in the time series in potential and current is greater in the AlSi10Mg alloy manufactured by additive manufacturing. The amplitude of the transients decreases in both alloys (AA6061 and AlSi10Mg) as time increases.

## 1. Introduction

The Layered Manufacturing about the nature of the process is additive manufacturing, where three-dimensional parts can be made [1,2,3].

Additive Manufacturing (A.M.) technologies have been developed to fabricate parts using metal powder. A.M. can be classified into two processes: (1) laser metal deposition (LMD), known as direct laser fabrication (DLF), (2) selective laser melting (SLM), selective electron beam melting (SEBM), or direct metal laser sintering (DMLS) [3,4].

Emerging layer-by-layer manufacturing technology for producing metallic components and parts is additive manufacturing by selective laser melting (SLM). This technique uses a laser to transform metallic powder into a solid piece [5,6,7]. The advantages of this technique are reduced consumption of raw materials and energy, rapid and continuous production, and the fabrication of geometrically complex parts [8,9]. The SLM process produces an unusual microstructure due to its rapid solidification. The microstructure differences change the mechanical and corrosion properties compared to components made by conventional methods [10,11].

AlSi10Mg alloy is applied in industries such as automotive and aerospace due to its low density, corrosion, and mechanical resistance [12]. Factors as differences in phases, segregation, or dissimilar grain sizes can affect the mechanical properties of the materials [13,14].

The corrosion resistance of Al-Si alloys manufactured by SLM is highly dependent on surface conditions. Having a rough surface can degrade both electrochemical performance and material life under dynamic loads. Furthermore, corrosion resistance is also related to the natural ability of the material to form a passive and adherent layer under regular atmospheric conditions [15,16].

Fathi et al. [2] studied the corrosive behavior in AlSi10Mg alloy manufactured by SLM in a 3.5 wt. % NaCl solution, similar to the seawater environment, compared to the A360.1 alloy manufactured by die casting. It was found that the material manufactured by MA exhibits greater resistance to corrosion than the material manufactured by die casting. The extra-fine microstructure included homogeneously distributed Si particles and an absence of intermetallic particles containing Fe and Cu. They are present in the A360.1 alloy.

The electrochemical noise (EN) technique is employed to study corrosion processes, mainly localized processes. Transients manifested in potential and current time series are related to different corrosion processes as localized re-passivation or pitting [17,18,19,20,21].

Ma et al. [22,23] mentioned that the surface area of working electrodes, symmetry, and electrolyte influence the measurement. Different methods can be employed to analyze corrosion processes. According to Xia et al. [24], time and the time-frequency domain as noise resistances and statistical parameters and power spectral density help identify corrosion rates and types. Additionally, alternative methods as the Hilbert–Huang transform and Wavelets can be used [24,25,26,27,28,29,30,31,32,33,34,35].

This research aimed to study the corrosion behavior of AA6061 and AlSi10Mg alloys produced by extruded and additive manufacturing, immersed at 3.5 wt. % in NaCl and H_2_O solutions at room temperature by electrochemical noise technique. Characterization by electrochemical techniques of aluminum alloys could find potential applications in the aeronautical industry as in fuselage and aircraft wings.

## 2. Materials and Methods

### 2.1. Materials

The materials used in this work were AA6061 and AlSi10Mg (Al-A.M) alloys. The chemical composition of the alloys was obtained by X-ray fluorescence (Olympus DELTA XRF. Richmond, TX, USA). Table 1 presents the chemical composition of each aluminum alloy.

The AlSi10Mg (Al-A.M) alloy was produced by additive manufacturing using selective laser melting (SLM). The printing parameters and metallurgical processing are reported in Table 2. The average diameter of the AlSi10Mg powder used (TLS Technik, Bitterfeld-Wolfen, Germany) was 38 µm (StDev 14 µm). The SLM manufacturing procedure was conducted by an SLM 280HL machine (Solutions GmbH, Hamburg, Germany). Bars were printed vertically, length = 100 mm and diameter = 10 mm.

### 2.2. Microstructural Characterization

The aluminum specimens were polished using metallographic techniques according to ASTM E3 [36]. The material was sequentially polished using different SiC grit papers with 400, 600, and 800 grades, followed by ultrasonic cleaning in ethanol (C_2_H_5_OH) and deionized water. The etching of polish samples was elaborated with a Kroll solution based on ASTM E 407 [37].

The microstructural analysis was carried out by optical microscopy (OM, Olympus, Hamburg, Germany) and scanning electron microscopy (SEM, JEOL-JSM-5610LV, Tokyo, Japan) for identifying the microstructure of samples a magnification of 500× and 1000× operating at 20 kV, WD = 11 mm. The chemical composition of these alloys was obtained by energy-dispersive X-ray Spectroscopy (EDS, Tokyo, Japan).

### 2.3. Corrosion Test

Electrochemical noise measurements were conducted at room temperature using potentiostat/galvanostat/ZRA Gill-AC from ACM Instruments (Manchester, UK).

A standard three-electrode cell was used composed of two nominally identical specimens used as the working electrodes (WE1 and WE2) and saturated calomel electrode (SCE) as reference electrodes [33,35,38]. Measurements were made at 0, 24, 48, and 120 h. For each experiment, 2048 data points were obtained with a scanning rate of 1 data/s [33].

The polynomial method was employed to remove the trend from EN signals and process statistical, PSD, and HHT information. To make an energy-disperse plot (EDP), an orthogonal wavelet transform was applied to the original signal (with DC) because this method separates the DC from the EN signal. EN analysis with the Hilbert–Huang transform (HHT) was necessary to obtain the intrinsic functions (IMF) of the EN signal by an empirical decomposition method (EMD). Finally, the instantaneous frequencies were plotted with a Hilbert spectrum. Data analysis was carried out with the MATLAB 2018a program (Math Works, Natick, MA, USA).

## 3. Results

### 3.1. Microstructural Analysis

**Figure 1:**
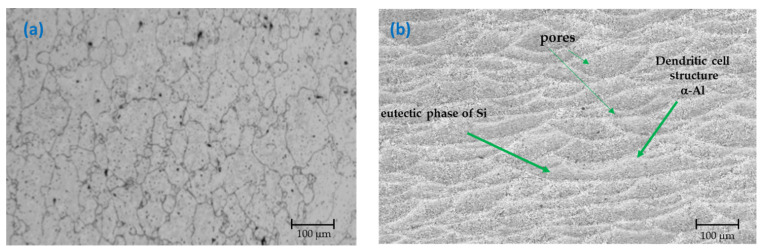
OM micrograph of alloys (initial conditions); (**a**) Al-6061 and (**b**) AlSi10Mg (Al-A.M).

The microstructures of the initial samples were analyzed by an optical microscope (OM). Figure 1a shows that in the Al-6061, the Al microstructure was in the α-Al matrix, which precipitated the Mg_2_Si. For the microstructure, it is evident that the eutectic was relatively coarse and discontinuous. This is attributed to the dissolution of eutectics in the solid solution during the solution treatment and the subsequent precipitation during aging (Figure 1b). The AlSi10Mg (Al-A.M) alloy matrix had a dendritic microstructure. The microstructure evolved due to the SLM manufacture, where the eutectic phase of silicon was observed and some pores of various sizes [16,39,40].

### 3.2. Electrochemical Noise

In the electrochemical noise signal, there are random, stationary, and continuous current variables. It is important to separate the DC signal from the aforementioned variables to analyze the EN data. The corrosion data, presented at low frequencies, were conserved [27,28,33,41,42,43,44].

The EN signal was filtered by a polynomial filter grade 5 to analyze only random and stationary components. The visual, statistical, PSD, and HHT analyses were made with the signal filter to obtain a study without false frequency and interference.

**Figure 2:**
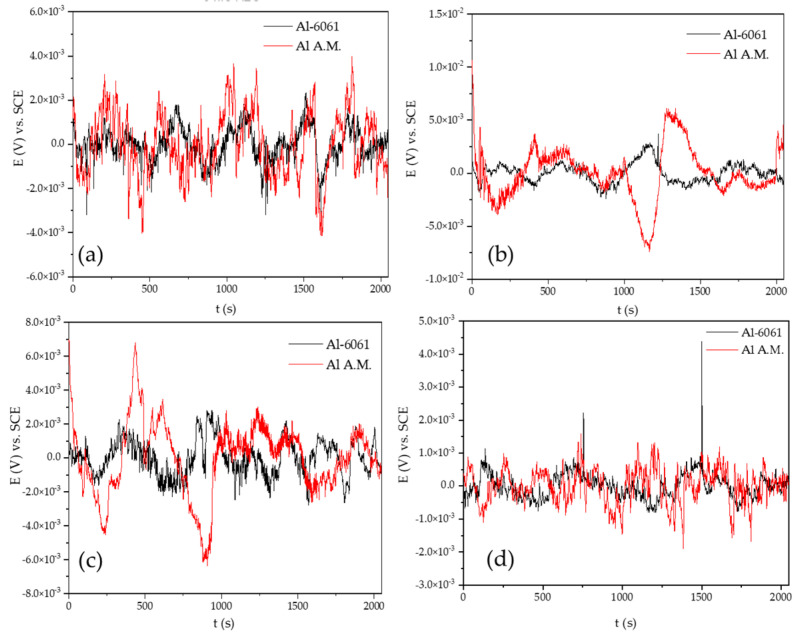
Time series electrochemical potential noise (EPN) data in H_2_O solution: (**a**) 0 h, (**b**) 24 h, (**c**) 48 h, and (**d**) 120 h.

**Figure 3:**
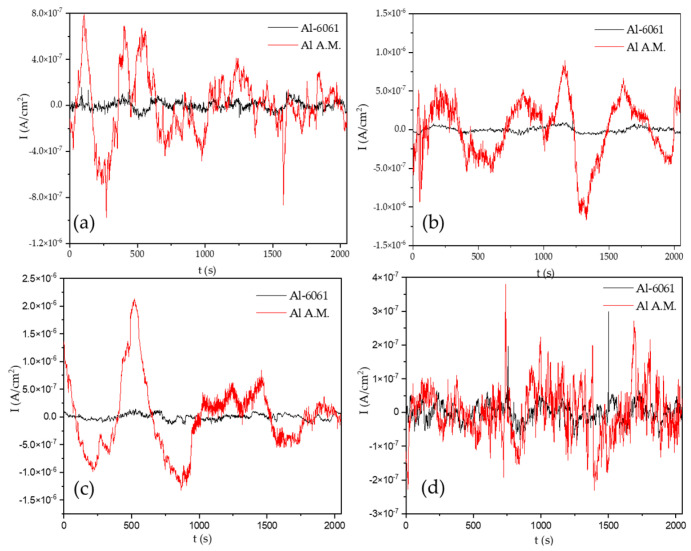
Time series electrochemical current noise (ECN) data in H_2_O solution, (**a**) 0 h, (**b**) 24 h, (**c**) 48 h, and (**d**) 120 h.

Figure 2 shows the EN signal in the potential for the Al-6061 and Al-A.M. samples in H_2_O at 0, 24, 48, and 120 h. The samples presented potentials in 10^−3^ orders. When samples were exposed for 0 h, the behavior was similar, but at 24, 48, and 120 h, Al-6061 decreased the amplitude; meanwhile, Al-A.M. increased the amplitude. Figure 3 shows the EN signal in the current at 0, 24, 48, and 120 h. Al-A.M. presented a higher amplitude of fluctuations than Al-6061, related to a faster corrosion kinetic. After 120 h of exposure, the Al-A.M. signal showed the presence of transients.

**Figure 4:**
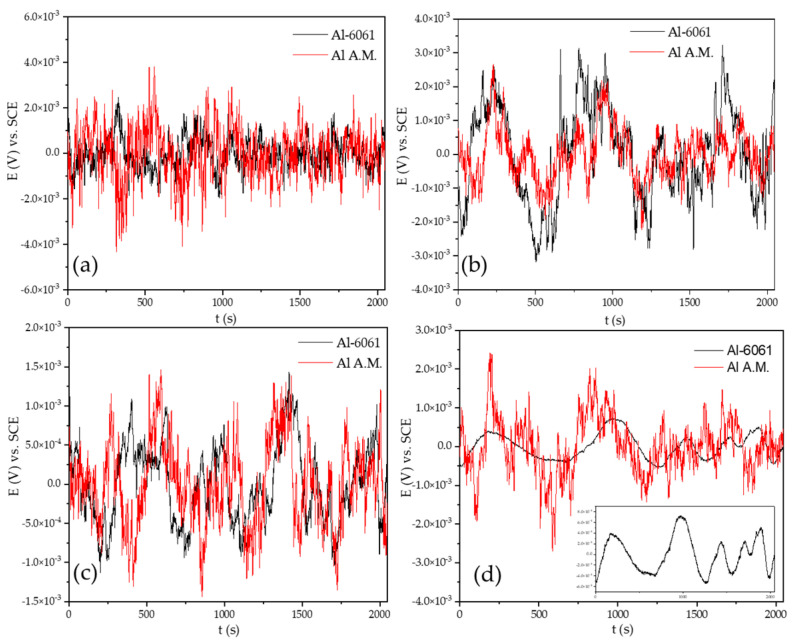
Time series electrochemical potential noise (EPN) data in NaCl solution. (**a**) 0 h, (**b**) 24 h, (**c**) 48 h, and (**d**) 120 h.

**Figure 5:**
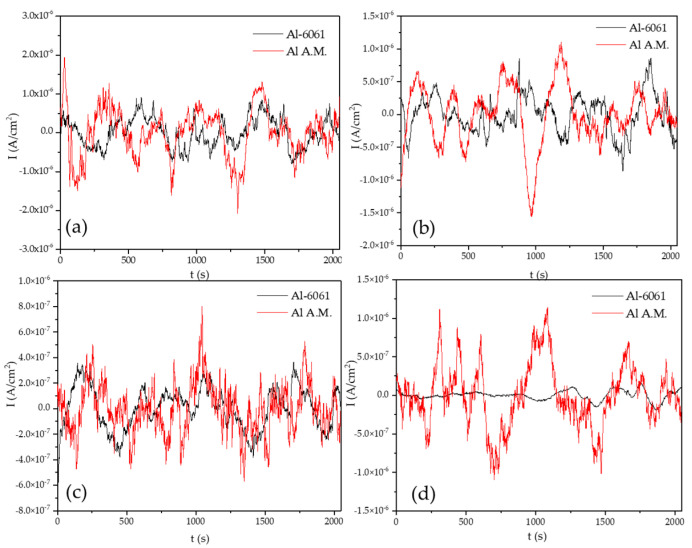
Time series electrochemical current noise (EPN) data in NaCl solution, (**a**) 0 h, (**b**) 24 h, (**c**) 48 h, and (**d**) 120 h.

Figure 4 shows the EN signal of the potential for the Al-6061 and Al-A.M. samples in NaCl at 0, 24, 48, and 120 h. Al-6061 and Al-A.M. presented 10^−3^ order transients at 0, 24, and 48 h. At 120, Al-A.M. presented higher fluctuations than Al-6061. Figure 5 shows the EN signal of the current in NaCl at 0, 24, 48, and 120 h. Both samples presented the same behavior at 0, 24, and 48 h, but after 120 exposition hours, Al-A.M. increased the current demand, related to higher corrosion kinetic than Al-6061.

The statistical analysis in Table 3 and Table 4 shows that the localization index (LI) had mixed and localized corrosion values, but skewness had values of uniform corrosion. The discrepancies in LI, skewness, and Kurtosis results could be related to the predominance of the uniform corrosion processes.

#### 3.2.1. Statistical Analysis

To obtain statistical parameters as *R_n_,* it is necessary to obtain the standard deviation, Equation (1). Those statistical parameters are related to the corrosion system [34,41].

(1) $$\sigma_{x}=\sqrt{\bar{x^{2}}}=\sqrt{\frac{1}{N}\overset{N}{\underset{i=1}{\sum }}{\left(x_{1}-\bar{x}\right)}^{2}\;\;\;\;\;\;\;}$$

where *x*_1_ is the EN signal’s values, $\bar{x}$ the average, and *n* is the number of pints in the recording. The signal employed can be of ECN or EPN.

Noise resistance (*R_n_*) is the ratio of the potential standard deviation to the current standard deviation related to the area of the sample (Equation (2)).

(2) $$R_{n}=\frac{\sigma_{v}}{\sigma_{I}}\times A$$

The values of *R_n_* and *R_p_* are considered homologous to the Stern–Geary equation [45], so values of *R_n_* can be used to determine the corrosion kinetic.

The *I_rms_* is obtained by Equation (3):

(3) $$r.m.s=\sqrt{Xn^{2}+\sigma^{2}}$$

The localization index (*LI*) is obtained by Equation (4).

(4) $$LI=\frac{\sigma_{i}}{I_{r.m.s}}$$

The values obtained are related to the corrosion type, according to diverse authors [32,33,34,46].

Also, Kurtosis and skewness could define the corrosion type. Equations (5) and (6) show how to obtain those parameters [47,48,49]:

(5) $$skewness=\frac{1}{N}\overset{N}{\underset{i=1}{\sum }}\frac{{\left(x_{i}-\bar{x}\right)}^{3}}{\sigma^{3}}$$

(6) $$kurtosis=\frac{1}{N}\overset{N}{\underset{i=1}{\sum }}\frac{{\left(x_{i}-\bar{x}\right)}^{4}}{\sigma^{4}}$$

Table 3 and Table 4 show *R_n_*, *i_corr_*, *skewness*, and *Kurtosis* from the EN signal after removing the DC signal with a 9^th^-grade polynomial.

High Kurtosis values can indicate instability or high amplitude transients in different distributions, provoking different processes on the metal surface [50].

#### 3.2.2. Power Spectral Density

With Equations (7) and (8), time-domain data is transformed to the frequency domain employing a Fast Fourier Transformation [51].

(7) $$R_{xx}\left(m\right)=\frac{1}{N}\sum_{n=0}^{N-m-1}x\left(n\right)\cdot x\left(n+m\right),\;\;\;\mathrm{when}\;\mathrm{values}\;\mathrm{are}\;\mathrm{from}\;0<m<n$$

(8) $$\Psi_{x}\left(k\right)=\frac{\gamma \cdot t_{m}}{N}\cdot \overset{N}{\underset{n=1}{\sum }}\left(x_{n}-{\bar{x}}_{n}\right)\cdot e^{\frac{-2\pi kn^{2}}{N}}$$

To evaluate the slope and the frequency zero limits (*Ψ^0^*), information about the corrosion mechanism and corrosion kinetic (respectively) Equation (9) can be applied [20,33,52,53]. It is important to clarify that only PSD in current gives information about material dissolution (*Ψ^0^*) [49,50,51,52,53,54].

(9) $$log\Psi_{x}=-\beta_{x}\log f$$

The frequency zero limits (*Ψ^0^*) give material dissolution information because PSD is related to the total energy present in the system [20,33,53]. It is essential to clarify that material dissolution is only present in the current PSD [49,50,51,52,53,54].

**Figure 6:**
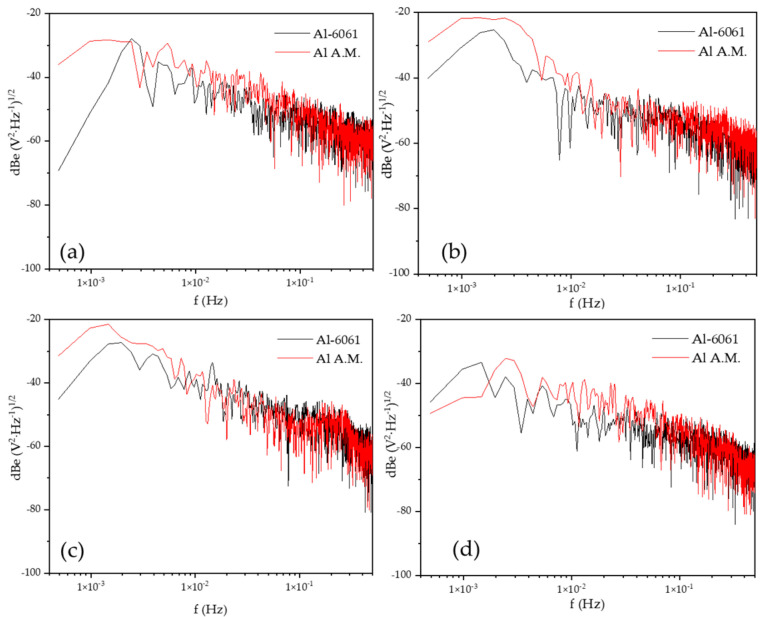
Power spectral density (PSD) of voltage in H_2_O solution, (**a**) 0 h, (**b**) 24 h, (**c)** 48 h, and (**d**) 120 h.

Figure 6 shows the PSD in voltage in H_2_O at different immersion times, dBe vs. f (Hz). The slope value (Β) in voltage did not fit with the values of the intervals (see Table 5). Figure 3a shows the behavior of stabilization, Figure 3b–d present fluctuations indicating a possible pitting process; the change of slope at high frequencies corresponded with a diffusion process related to the diffusion of pitting.

**Figure 7:**
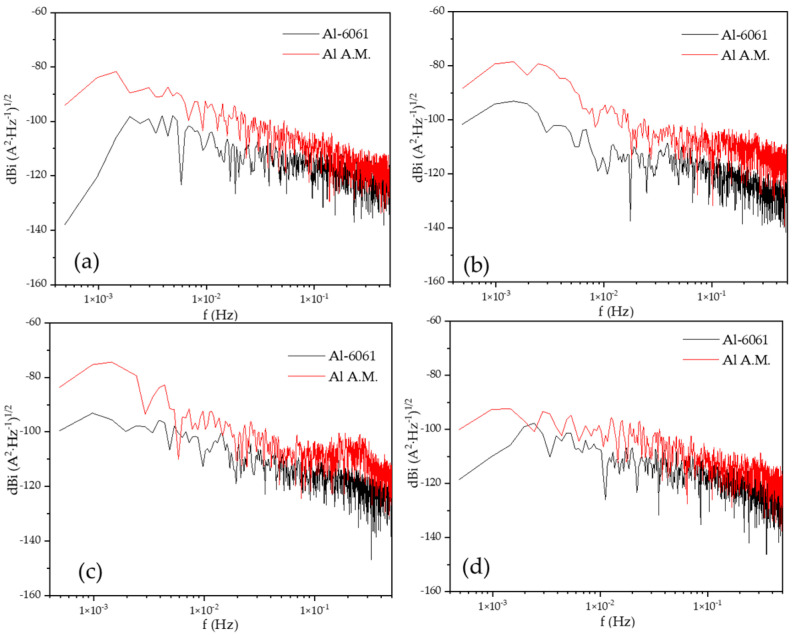
Power spectral density (PSD) in the current in H_2_O solution, (**a**) 0 h, (**b**) 24 h, (**c**) 48 h, and (**d**) 120 h.

Figure 7 shows the PSD in the current of H_2_O at different immersion times, dBi vs. f (Hz). The current slope value (B) was related to the pitting process in all the immersion times (see Table 6). The value of *ψ^0^* was higher for the Al-A.M. samples than for conventional aluminum (see Table 6), related to the high corrosion kinetic for Al-A.M. samples.

**Figure 8:**
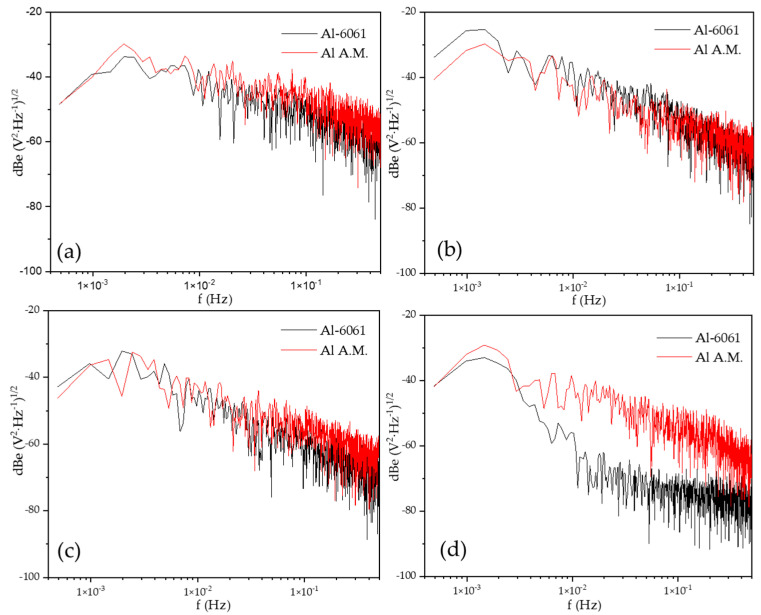
Power spectral density (PSD) in voltage in NaCl solution, (**a**) 0 h, (**b**) 24 h, (**c**) 48 h, and (**d**) 120 h.

**Figure 9:**
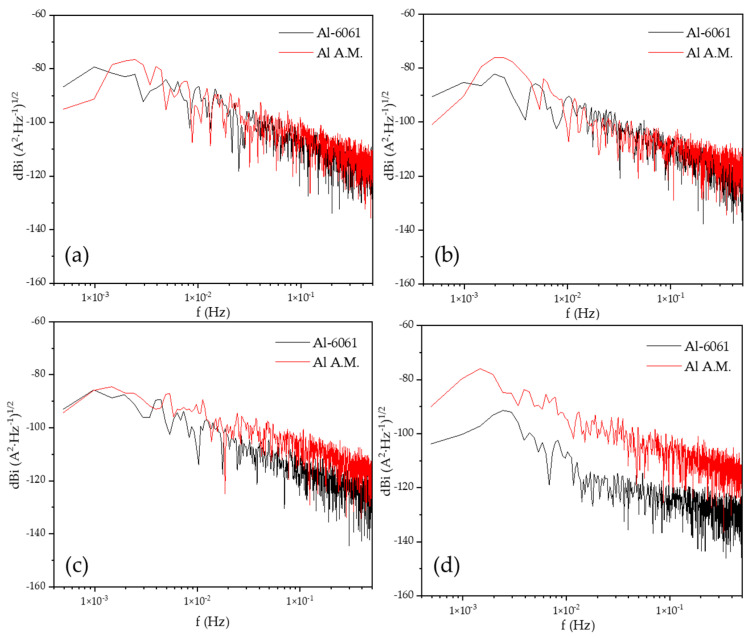
Power spectral density (PSD) in current in NaCl solution, (**a**) 0 h, (**b**) 24 h, (**c**) 48 h, and (**d**) 120 h.

Figure 8 shows the PSD in voltage in NaCl at different immersion times, dBe vs. f (Hz). The Al-A.M. and Al-6061 samples showed similar behavior at 0, 24, and 48 h, but at 120 h, the Al-A.M. sample presented higher values than Al-6061 and higher slope (see Table 6). Figure 9 shows the PSD in the current in NaCl at different immersion times, dBi vs. f (Hz). Slope values corresponded to the pitting process for both samples (see Table 5 and Table 6). The *ψ^0^* value was higher for Al-6061 at 0, 24, and 48 h, but at 120, Al-A.M. presented a higher value. The increase in the *ψ^0^* value indicated that Al-A.M. increased its corrosion kinetics concerning the time of exposure in NaCl. Additionally, Al-6061 showed a decrease in *ψ^0^*, meaning a stabilization in the electrolyte.

#### 3.2.3. Noise Impedance (*Z_n_*)

The noise impedance, *Zn* (f), also called spectral noise resistance, is defined as [50,55]:

(10) $$Z_{n}=\sqrt{\frac{\psi_{V}\left(f\right)}{\psi_{I}\left(f\right)}}$$

*Zn* is calculated by the square root of the PSD division of potential and current [33,41,55]. The electrochemical noise impedance is related to the corrosion resistance, and the inverse is related to the conductance and corrosion rates [37,56].

**Figure 10:**
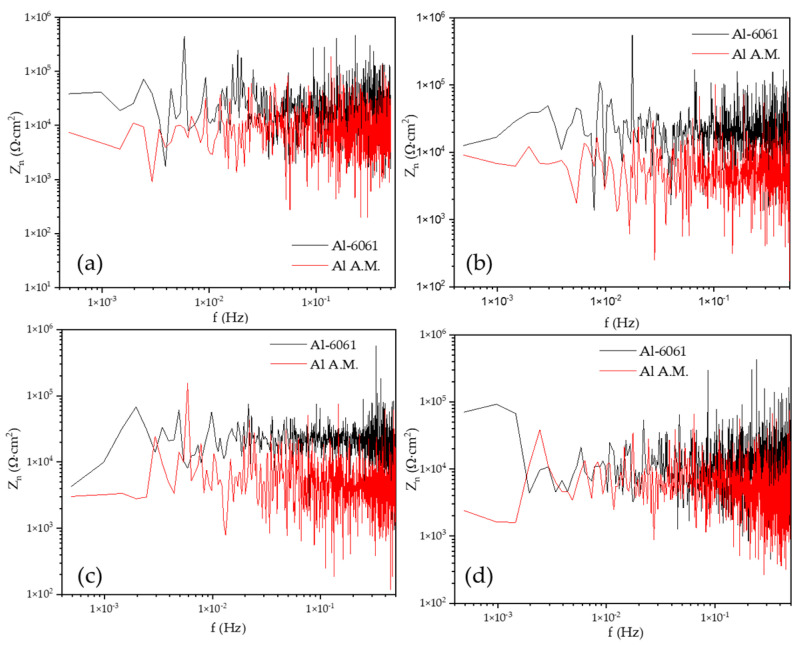
Noise impedance in H_2_O solution, (**a**) 0 h, (**b**) 24 h, (**c**) 48 h, and (**d**) 120 h.

Figure 10 presents the noise impedance (*Z_n_*) in H_2_O. The behavior of Al-A.M. samples corresponded with the values obtained in ψ^0^. The Al-A.M. samples presented the lower values of Z_n_0 (see Table 6), associated with lower corrosion resistance. In the whole graph, Al-6061 presented higher values of Z_n_.

**Figure 11:**
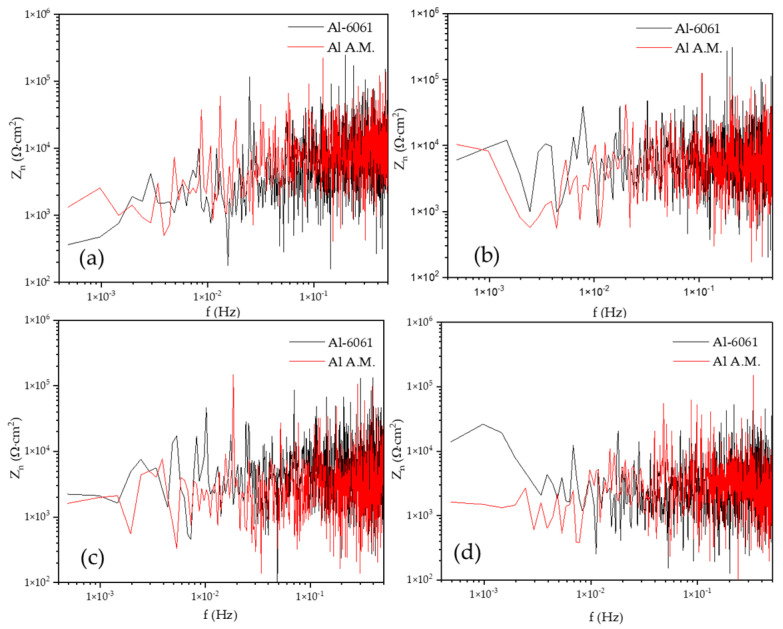
Noise impedance in NaCl solution, (**a**) 0 h, (**b**) 24 h, (**c**) 48 h, (**d**) 120 h.

Figure 11 shows the noise impedance (Z_n_) vs. f (Hz). The behavior for Z_n_0, rather than for ψ^0^, and the noise impedance of Al-6061 increased after 48 h (see Table 6) of exposition in NaCl, meaning an increase in corrosion resistance of this alloy.

#### 3.2.4. Wavelet Method

Wavelets methods decompose a signal with a high–low filter: low frequencies are named approximations, and high frequencies are called details [43,57,58]. To obtain the total energy of an N number of data, Equation (11) is employed [34]:

(11) $$E=\overset{N}{\underset{n-1}{\sum }}x_{n}^{2}$$

Furthermore, energy fractions of details and approximation are giving by Equation (12):

(12) $$ED_{j}^{d}=\frac{1}{E}\overset{N}{\underset{n=1}{\sum }}d_{j,n}^{2}\;\;ED_{j}^{s}=\frac{1}{E}\overset{N}{\underset{n=1}{\sum }}s_{j,n}^{2}$$

The total energy analyzed is equal to each component energy of the wavelet transform, Equation (13):

(13) $$E=ED_{j}^{s}\overset{j}{\underset{j=1}{\sum }}ED_{j}^{d}$$

For this research, the number of crystals to analyze was eight details and one approximation. When energy was accumulated on the first crystals (D1–D3), it was attributed to a metastable pitting process. When major energy was presented on the crystals from D4 to D6, it was associated with localized corrosion; if energy was present in crystals D7 and D8, it was due to diffusion, a generalized or controlled process [43,51,59]. Crystal S8 (approximation) was related to the DC from the EN signal. Equation (16) must be applied to determine each crystal time [60]:

(14) $$\left(c_{1}^{j},c_{2}^{j}\right)=\left(2^{-j}\Delta t,2^{j-1}\Delta t\right)$$

where *c* is a crystal and *∆t* is the time display. High-frequency crystals are the first, and low-frequency phenomena are presented in the last crystals.

**Figure 12:**
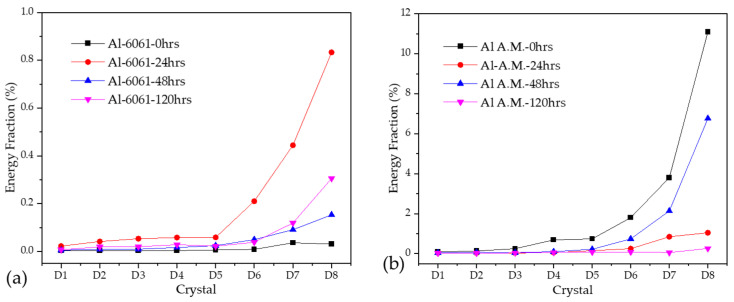
Wavelets in the H_2_O solution (**a**) Al-6061 and (**b**) Al-A.M.

Figure 12 shows the EDP from the ECN signal in H_2_O. For both alloys, the maximum energy accumulation was found in crystals D7 and D8. However, Al-A.M. presented a higher percentage of energy accumulation at 0 h of exposure, decreasing at 24, 48, and 120 h. The higher energy accumulation at 0 h was related to the high porosity of the samples. At 0 h, an ionic diffusion in porous occurred. Both samples presented a diffusion of pitting.

**Figure 13:**
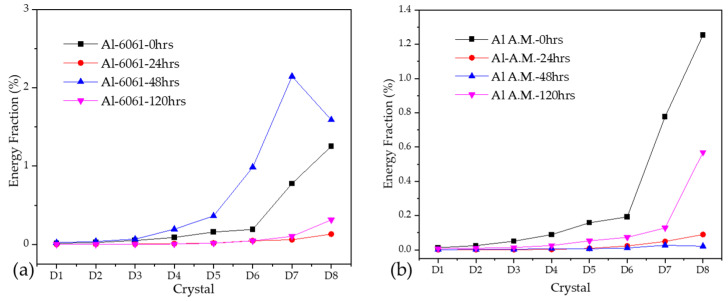
Wavelets in NaCl solution (**a**) Al-6061 and (**b**) Al-A.M.

Figure 13 shows the EDP from the ECN signal in NaCl. The Al-6061 in Figure 12a presented a higher energy accumulation after 48 h, with energy at crystals D4 to D8. The presence of the energy at intermedium crystals was related to a possible diffusion of pitting. The Al-A.M. sample presented a similar behavior in H_2_O, where high porosity presence produced an ionic diffusion. After 120 h of exposure, the Al-A.M. sample presented an increase in energy accumulation related to the possible diffusion of pitting by Cl^−^ ions.

#### 3.2.5. Hilbert–Huang Transform Analysis

Another advanced method to determine the corrosion type and mechanism is HHT, which helps remove DC from the original signal [61]. In addition, this method can localize the frequency and time when the energy interchange is occurring. The energy is called instantaneous energy and is calculated by an empirical method of decomposition (EMD) to obtain intrinsic functions (IMF) and apply HHT as proposed by Huang et al. [62] to study non-stationary signals. A spectrum with time-frequency energy distribution is generated, permitting localized energy to accumulate [33,46,63,64,65]. EMD, proposed by Huang, is expressed in Equation (15):

(15) $$x\left(t\right)=\overset{N}{\underset{i=1}{\sum }}h^{\left(i\right)}\left(t\right)+d\left(t\right)$$

*d*(*t*) is the average of the trend at a low frequency of the time series *x*(*t*) and cannot be decomposed. *h*^(*i*)^(*t*) is the *i*^th^ term of IMF that is generated. The numbers must satisfy the conditions that the extreme and cross numbers are equal or differ by a maximum of 1 and that each point using the local maximum and minimum must be 0 [49,57,63]. The HHT Equation (16) is governed by:

(16) $$y_{j}\left(t\right)=\frac{1}{\pi }p\int_{-\infty }^{\infty }\frac{h_{j}\left(\tau \right)}{t-\tau }d\tau$$

where *y_j_*(*t*) is the Hilbert transform and IMF are represented with *h_j_*; *p* is related to the Cauchy principle and is linked with an average of IMF [60].

**Figure 14:**
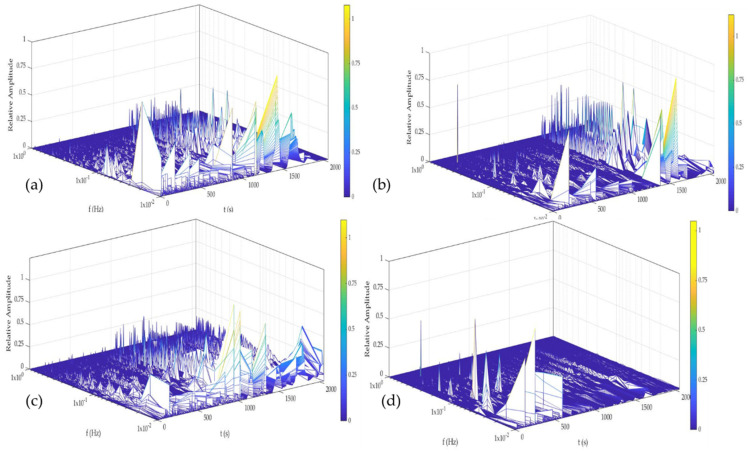
Hilbert spectra of Al-6061 in H2O solution (**a**) 0 h, (**b**) 24 h, (**c**) 48 h, and (**d**) 120 h.

**Figure 15:**
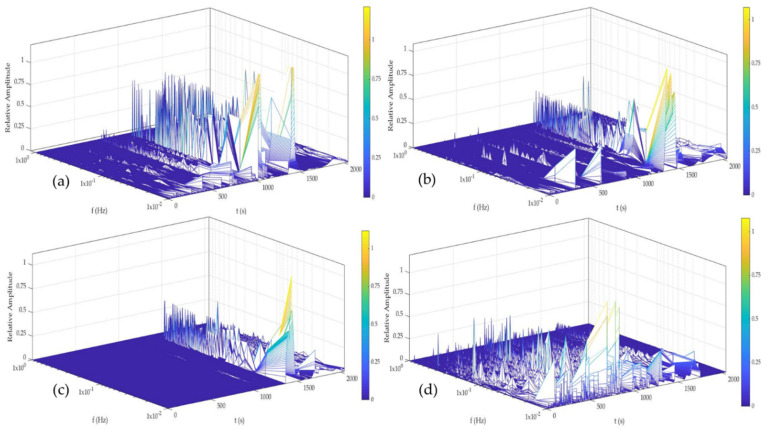
Hilbert spectra of Al-A.M. in H_2_O solution (**a**) 0 h, (**b**) 24 h, (**c**) 48 h, and (**d**) 120 h.

Figure 14 and Figure 15 show the time–frequency–energy spectra generate by HHT in H_2_O. Al-6061 presented a few energy accumulations at high and medium, and the maximum energy was localized at low frequencies at 0, 24, and 48 h. The accumulation of energy related to the pitting diffusion is shown in Figure 14d, where energy was present only at low frequencies. Al-A.M. presented energy accumulation at high and low frequencies at all times but with high energy at low frequencies (1 × 10^−2^ Hz). Al-A.M.’s behavior was related to the porosities that predisposed the alloy to pit.

**Figure 16:**
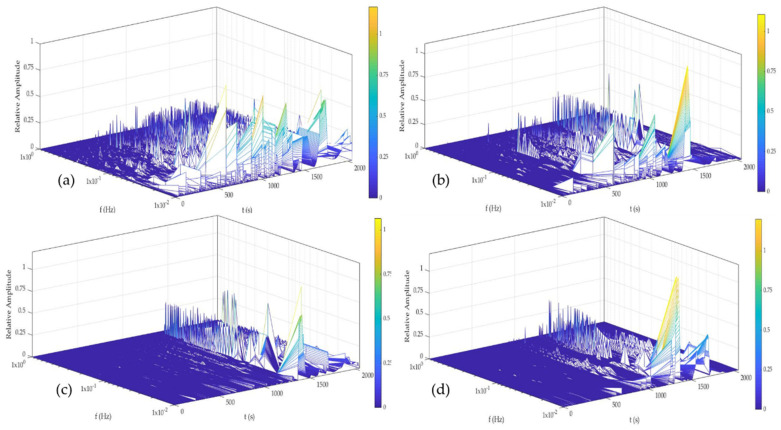
Hilbert spectra of Al-6061 in NaCl solution (**a**) 0 h, (**b**) 24 h, (**c**) 48 h, and (**d**) 120 h.

**Figure 17:**
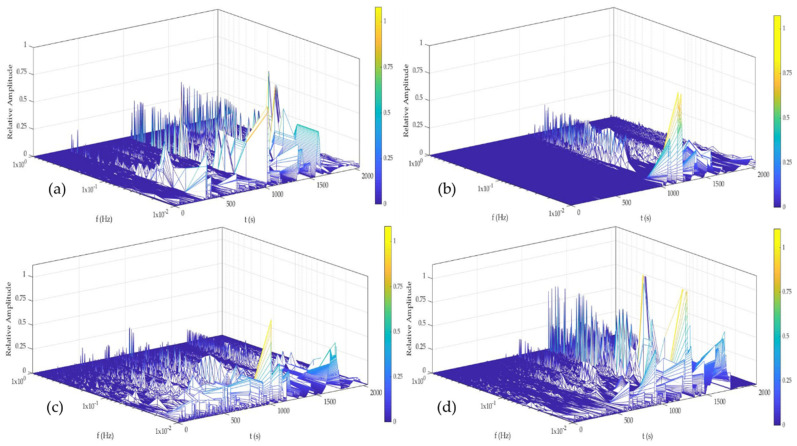
Hilbert spectra of Al-A.M. in NaCl solution (**a**) 0 h, (**b**) 24 h, (**c**) 48 h, and (**d**) 120 h.

Figure 16 and Figure 17 show the time–frequency–energy spectra generated by HHT in NaCl. Al-6061, in Figure 16, presented a behavior related to the diffusion of pitting. The sample presented energy at high and low frequencies (1 × 10^0^ and 1 × 10^−1^ Hz) but increased a low frequency (1 × 10^−2^). The behavior at 0 and 24 h was related to the process of high frequencies being (pitting) diffused. At 48 and 120 h (Figure 16c,d), the behavior was similar and associated with the pitting diffusion at 0 and 24 h. Al-A.M. showed (Figure 17a–d) a behavior of pitting diffusion.

#### 3.2.6. SEM Corrosion Product Analysis

**Figure 18:**
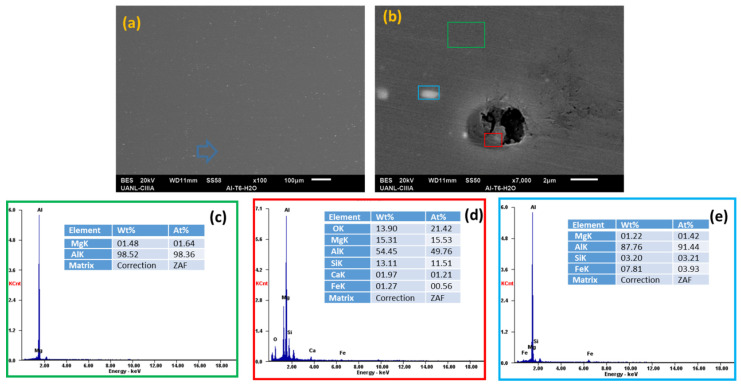
SEM-EDS surface micrographs of Al-6061 (**a**,**b**) alloys in H_2_O solution and EDS spectrum analysis: (**c**) green box, (**d**) red box, and (**e**) blue box.

**Figure 19:**
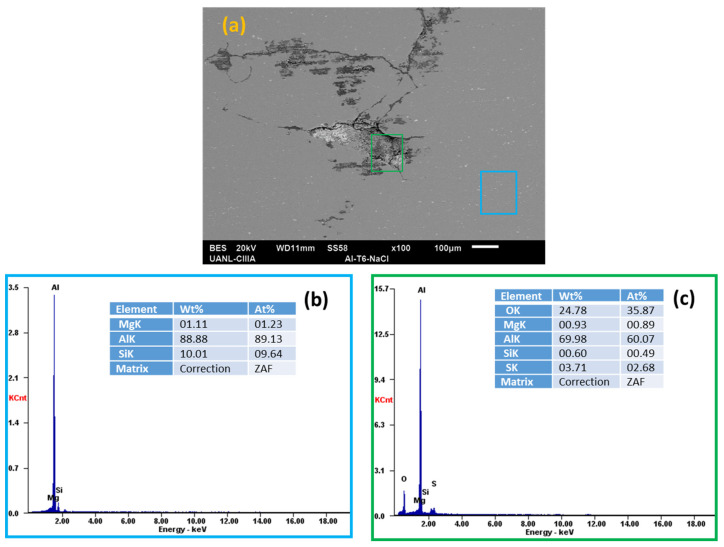
SEM-EDS surface micrographs of Al-6061 (**a**) alloys in NaCl solution and EDS spectrum analysis: (**b**) blue box and (**c**) green box.

**Figure 20:**
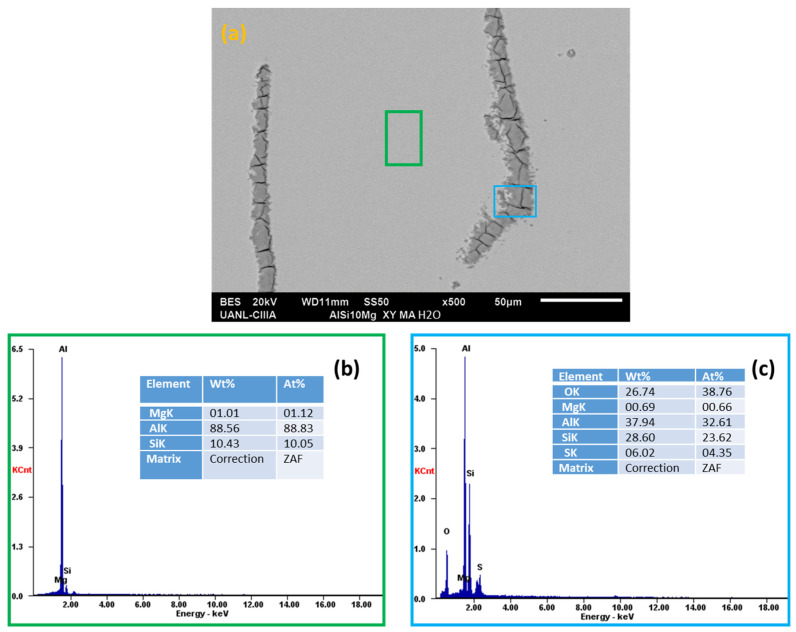
SEM-EDS surface morphology micrographs of Al-A.M. (**a**) alloys in H_2_O solution and EDS spectrum analysis: (**b**) green box and (**c**) blue box.

**Figure 21:**
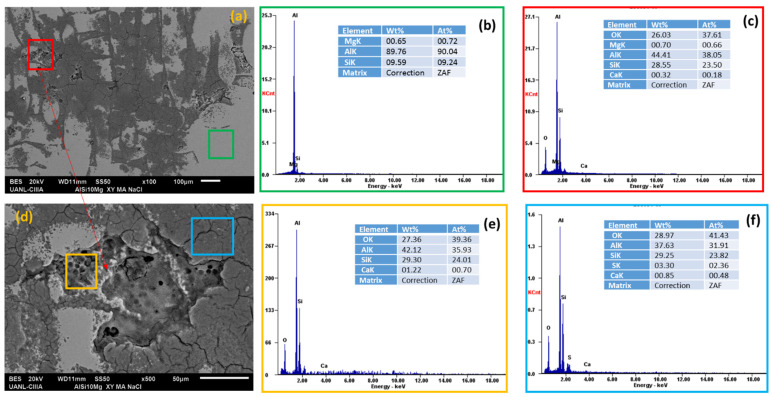
SEM-EDS surface morphology micrographs of Al-A.M. (**a**,**d**) alloys in NaCl solution and EDS spectrum analysis: (**b**) green box, (**c**) red box, (**e**) orange box, and (**f**) blue box.

Scanning electron microscopy (SEM) and energy-dispersive X-ray spectroscopy (EDS) analyzed the morphology of the aluminum alloys and the elements presented on the surface after the electrochemical experiments, see Figure 18, Figure 19, Figure 20 and Figure 21.

In the EDS energy spectrum, aluminum, magnesium, and silicon were observed to be corresponding to the base elements of the alloys under study.

Aluminum alloys in H_2_O and NaCl solutions (Figure 18a,b, Figure 19, Figure 20 and Figure 21a,b) did not have oxygen, but there was corrosion and pitting products in some cases. When the AA6061 alloy was in contact with water (Figure 18d), it had small pits of about 2 microns. The presence of oxygen was indicated in the red box (average 13.90 wt. %), Figure 19c, the spectra EDS indicated the presence of oxygen and silicon in the darker zone, marked with a blue box (average 20.36 and 28.60 wt. %), respectively [16].

The Al-6061 and Al-A.M. alloys exposed to sodium chloride presented corrosion products (Figure 19 and Figure 21) with more severe corrosion, having a greater presence of oxygen in the grain boundary zones for Al-A.M. alloys.

## 4. Discussion

The corrosion resistance of aluminum and its alloys depends on the chemical composition of the material. The alloying elements determine the mechanical and corrosion resistance properties of these alloys.

Microstructural analysis indicated that alloys produced by additive manufacturing- selective laser melting were more susceptible to localized corrosion (see results skewness, Kurtosis, and localization index). The porosity of A.M. alloys was a compromise between mechanical strength and adequate pore size to obtain specific operating properties [52,66,67,68]. Pores are stress concentrators; Seah et al. [69] concluded that porosity makes material susceptible to localized corrosion. Nevertheless, an increase in the porosity of metals led to a lower corrosion potential value, which resulted in increased susceptibility of porous materials to localized corrosion. The microstructure obtained by the A.M. process presented differences from the conventional process (e.g., extruded). The microstructures of A.M. materials can present non-uniform structures (dendritic microstructure), affecting the electrochemical behavior of the material [70].

The microstructure obtained by the A.M process presented differences from the conventional process. The microstructures of the A.M. materials can present non-uniform structures, affecting the electrochemical behavior of material [71]. The materials manufactured by A.M. presented a poor passive layer. For the Al-alloys studied in this research, the non-uniform passive layer created induced the pitting. This behavior was related to a non-uniform microstructure. Some cases reported that the defecting passive layer promoted crevice corrosion. Additionally, cathodic reactions generated hydrogen and exposed the alloy to HIC. HHT results showed how a weak, passive layer was created; even in H_2_O and NaCl. The energy presented at high and medium frequencies (Figure 15 and Figure 17) was associated (particularly in Figure 15b–d and Figure 17b–d) with localized attacks due to a non-homogenous passive layer. For the characteristic of the poor passive layer, the localized attacks began to diffuse after a time, so high energy was presented at low frequencies indicating pitting diffusion. Other authors such as Lin et al. [72,73] commented that in order to improve the corrosion resistance of light alloys it is important to consider the effect of precipitate pretreatments on the microstructure, seeking to ensure that they are well distributed. The concentration of precipitates of Cu, Zn, or Mg can cause cracks or failures in the alloys, and this was reflected in the transients of the electrochemical noise-generating drops in potential and increase in the current [72,73].

Pitting diffusion was also attributed to Cl^-^ ions attack. Authors such as Chiu et al. [74] related the Cl^-^ ions attack with the deterioration of the passive layer in A.M. materials. Interstitial ions deteriorated the oxide layer, and an aggressive ion penetration and dissolution occurred on the surface. Further, an adsorption phenomenon can occur on the surface metal. Azar et al. [75] found a connection for chloride adsorption on alumna surface film selecting in different interfaces between precipitates. The alumina film was soluble in alkaline pHs generated in oxygen reduction, dissolving the non-uniform passive layer. The oxygen reduction occurred in anodic reactions, which were visible in time series and HHT. When anodic transients presented a higher oxygen reduction, a dissolution of material occurred in high-energy transients. As high-energy transients occurred at low frequencies, it can be related in PSD with the events occurring in ψ^0^, where a material dissolution happened. Microstructure properties as precipitates induced the autocatalytic process, and the authors Melia et al. [76] associated the precipitates with an increase in the corrosion process. Further, Kubacki et al. [77] observed an aluminum dissolution due to a discontinuous silicon melt, and Revilla et al. [78] also attributed a poor melting process with the corrosion susceptibility of alloys.

Furthermore, Xu et al. [79] observed a non-uniform passive layer develop when the material was exposed to NaCl at 3.5%. The material presented surface exfoliation and pitting corrosion and related the corrosion resistance to the homogeneity of the grain refinement of microstructure. This research presented the same behavior, where grain was not homogenous, and SEM-EDS images showed surface exfoliation and pitting that occurred on the surface.

The non-homogenous passive layer was also related to surface defects associated with the manufacturing process inducing a pitting attack [80]. Sander et al. [81] attributed the repassivation problems to the porosity of additive manufacturing samples. Additionally, the results suggested that scan speed and laser power did not play an important factor. Defects such as porosity can be identified by the visual and HHT method. In visual analysis, the transients presented in Figure 2, Figure 3, Figure 4 and Figure 5 at 0 immersion hours were higher for A.M. than for conventionally manufactured samples. HHT method showed in Figure 15a and Figure 17a that there was high activity in energy at high and medium frequencies (from 0.5 to 0.75) compared to the conventionally manufactured sample (0.25 to 0.4). The porosities increased the anodic-cathodic process occurring on the surface; therefore, HHT (Figure 15a and Figure 17a) presented a higher energy diffusion at higher and medium frequencies. The cathodic-anodic process was fast, so electrochemical noise is a powerful technique to determine the homogeneity of metal surfaces in the first seconds of the test.

Some authors [81,82,83,84,85,86] suggested applying heat treatments to reduce this class of defects and increase microstructure uniformity. This way, corrosion resistance increased because when the microstructure is uniform, corrosion pitting or a non-uniform passive layer is reduced. Additionally, post-heat treatment in AlSi10Mg alloys with temperatures between 200 and 300 °C reported good results. When the temperature increased to 400 °C, the formation of Mg_2_Si precipitates increased the probability of a localized attack because Si acted as a cathode. This information helped to determine future works of this research by applying heat treatments to A.M. samples.

## 5. Conclusions

In this work, characterization by electrochemical noise of Al-6061 and Al-A.M. (AlSi10Mg) alloys produced by extruded and additive manufacturing could find potential applications in the aeronautical industry.

- Microstructural analysis indicated that alloys produced by additive manufacturing- SLM were more susceptible to localized corrosion due to porosity.
- EN results showed that the amplitude of the transients in both the potential and current time series was greater in the AlSi10Mg (Al-A.M.) alloy manufactured by additive manufacturing.
- The localization index, skewness, and Kurtosis results showed that they must be interpreted to measure the disorder and distribution of transients and not as a mechanistic method for aluminum alloys.
- EN results showed that Z_n_ and Ψ^0^ parameters should be considered a counterpart to calculate the corrosion resistance of materials.
- Wavelets and HHT methods were more reliable in determining the corrosion type for Al-6061 and Al-A.M. alloys than statistical methods. In H_2_O and NaCl, wavelets and HHT presented similar results. For NaCl, the behavior was associated with a slow process, but the energy presence at middle frequencies was significant, and an unstable passive layer was attributed to Cl^−^ ions.
- SEM-EDS observations indicated that Al-6061 and Al-A.M. alloys exposed to sodium chloride presented corrosion products with more severe corrosion, having a greater presence of oxygen in the grain boundary zones for Al-A.M. alloys.

## Figures and Tables

**Table 1:** Chemical composition of the used aluminum alloys (wt. %).

Alloy	Si	Fe	Cu	Mg	Zn	Ti	Cr	Mn	Al
AA6061-T6	0.6 ± 0.03	0.7 ± 0.35	0.18 ± 0.01	0.9 ± 0.045	0.25 ± 0.013	0.015 ± 7.5 × 10^−4^	0.35 ± 0.018	-	Bal.
AlSi10Mg (A.M)	10.1 ± 0.5	0.16 ± 0.008	0.001 ± 5 × 10^−5^	0.35 ± 18 × 10^−4^	0.002 ± 1 × 10^−4^	0.01 ± 5 × 10^−4^	-	0.002 ± 1 × 10^−4^	Bal.

**Table 2:** SLM processing parameters are used to manufacture aluminum alloys.

Parameter	Value
Laser power, W	400
Scanning speed, mm/s	230
Layer thickness, µm	30
Hatch spacing, µm	110
Energy density, J/mm^3^	527
Scan rotation between successive layers	90°

**Table 3:** EN statistical parameters for Al-6061 alloys and electrolytes.

H_2_O Solution								
Time (h)	*R_n_* (Ω·cm^2^)	*i_corr_* (mA/cm^2^)	LI	Corrosion Type	Skewness	Corrosion Type	Kurtosis	Corrosion Type
0	19,405.77± 970	1.3 × 10^−6^ ± 6.5 × 10^−8^	0.032	Mix	0.17	Uniform	2.67	Uniform
24	25,422.38 ± 1271	1.0 × 10^−6^ ± 5.0 × 10^−8^	0.131	Localized	0.27	Uniform	2.33	Uniform
48	20,344.82 ± 1017	1.2 × 10^−6^ ± 6.0 × 10^−8^	0.051	Mix	−0.008	Uniform	2.55	Uniform
120	13,476.44 ± 674	1.9 × 10^−6^ ± 9.5 × 10^−8^	0.044	Mix	0.81	Uniform	11.31	Uniform
**NaCl Solution**								
0	1810 ± 90	14.4 × 10^−6^ ± 7.2 × 10^−7^	0.044	Mix	−0.002	Uniform	2.23	Uniform
24	4881.08 ± 244	9.1 × 10^−6^ ± 4.5 × 10^−7^	0.056	Mix	0.037	Uniform	3.00	Uniform
48	3021.06 ± 151	8.6 × 10^−6^ ± 4.3 × 10^−7^	0.27	Localized	−0.3	Uniform	2.93	Uniform
120	5277.3 ± 263	4.9 × 10^−6^ ± 2.5 × 10^−7^	0.055	Mix	−0.45	Uniform	3.82	Mix

**Table 4:** EN statistical parameters from Al-A.M. alloys and electrolytes.

H_2_O Solution								
Time (h)	*R_n_* (Ω·cm^2^)	*i_corr_* (mA/cm^2^)	LI	Corrosion Type	Skewness	Corrosion Type	Kurtosis	Corrosion Type
0	5145.31 ± 257	5.1 × 10^−6^ ± 2.6 × 10^−7^	0.344	Localized	0.046	Uniform	3.39	Localized
24	6547.6 ± 327	4.0 × 10^−6^ ± 2.0 × 10^−7^	0.17	Localized	−0.46	Uniform	2.99	Uniform
48	3358.78 ± 168	7.7 × 10^−6^ ± 3.9 × 10^−7^	0.051	Mix	0.71	Uniform	2.55	Localized
120	6800.02 ± 340	5.7 × 10^−6^ ± 2.9 × 10^−7^	0.26	Localized	0.26	Uniform	4.02	Uniform
**NaCl Solution**								
0	1810 ± 90	14.4 × 10^−6^ ± 7.2 × 10^−7^	0.044	Mix	−0.002	Uniform	2.23	Uniform
24	4881.08 ± 244	9.1 × 10^−6^ ± 4.5 × 10^−7^	0.056	Mix	0.037	Uniform	3.00	Uniform
48	3021.06 ± 151	8.6 × 10^−6^ ± 4.3 × 10^−7^	0.27	Localized	−0.3	Uniform	2.93	Uniform
120	5277.3 ± 263	4.9 × 10^−6^ ± 2.5 × 10^−7^	0.055	Mix	−0.45	Uniform	3.82	Mix

**Table 5:** Parameters obtained by PSD for the Al-6061 alloy.

Al-6061 Alloy				
Time (h)	*Ψ^0^* (dBi)	Z_n_0 (Ω·cm^2^)	Β (dB (V))	Β (dB (A))
H_2_O				
0	−138.01	38,612.3	−9.5	−8.4
24	−101.59	12,627.58	−11.4	−11.1
48	−99.61	4252.54	−11.7	−10.8
120	−118.61	70,334.05	−9.4	−9.5
NaCl				
0	−86.81	363.47	−9.7	−14.8
24	−90.53	6027.83	−14.8	−15.3
48	−93.04	2217.93	−12.6	−14.2
120	−103.81	13,962.53	−8.7	−10

**Table 6:** Parameters obtained by PSD for the Al-A.M. alloy.

Al-A.M.—Alloy				
Time (h)	Ψ^0^ (dBi)	Z_n_0 (Ω·cm^2^)	Β (dB (V))	Β (dB (A))
H_2_O				
0	−94.11	7473.53	−13.1	−13.1
24	−88.42	9077.61	−10.6	−10.2
48	−83.69	3030.07	−12.1	−10.3
120	−100.11	2410.34	−13.8	−10.8
NaCl				
0	−95.09	1320.19	−9.5	−13.5
24	−100.81	10,368.45	−10.2	−11.4
48	−94.37	1609.84	−10.6	−12.1
120	−90.08	1629.51	−11.9	−11.6

## Data Availability

The data presented in this study are available on request from the corresponding author.
